# What It Has Come to in Wales

**Published:** 1913-12-27

**Authors:** 


### THE PROFESSION OF MEDICINE.

### What it Has Come to in Wales.

Many parts of the United Kingdom have special peculiarities in so far as medical practice is concerned, due to the local characteristics of the district; and this applies to industrial and contract practice perhaps with even greater force than to private practice of the old-fashioned type. In Wales the developments of industrialism and trade unionism, combined with the peculiarities of the Welsh people, have tended to accentuate the differences between contract practice there and in England: at least in respect of the mining and manufacturing districts which contain so large a proportion of the inhabitants of Wales. In South Wales the result of these factors, together with the operation of the Insurance Act, has been to produce a condition of things which is degrading and derogatory to the medical practitioners there, and must be thoroughly bad business for the manual workers and their families as well. There can be little hesitation in pronouncing the medical arrangements in some of the colliery districts of Wales to be a national scandal, and a grave menace to the health and happiness of the whole population there. These evils are not wholly ascribable to the Insurance Act, which did not sow the seed, but merely watered it. The Insurance Commissioners for Wales appear, however, to have been culpable abettors of developments of the most deplorable kind; mainly, it may be surmised, from political motives, or at least through political inspiration.

Before proceeding to give chapter and verse for these formidable indictments, it may be premised that even that long-suffering body the British Medical Association cannot stomach the breaches of faith which have taken place, and has been constrained to pass a motion condemning the abuses in question. The terms of the resolution are: "That in order to check the extension of medical-aid institutions, and to assist the profession in combating them where established, the association endeavour to enlist the support of the staffs of voluntary hospitals not only in refusing professional recognition to the medical officers of these institutions, but also by refusing treatment to patients sent by them to hospitals, except in cases of grave urgency."

Let us take the example of Briton Ferry to illustrate these points. The situation at this mining town is graphically described in the *Lancet*, and it is horrible to contemplate. Admittedly, it is one of the worst cases, if not the very worst; but even feeble and far-off imitations of Briton Ferry would be a public danger and nuisance. The town contains about 9,000 inhabitants: about 3,000 of these are insured and the rest are almost entirely their dependants. Some 2,000 of the workers are members of an association known as the "Briton Ferry Workmen's and General Institution for Medical and Surgical Treatment." This has been officially recognised by the Welsh Insurance Commission, flat against the promises made by the Chancellor of the Exchequer to the British Medical Association. The result is that the moneys allotted out of Insurance Funds for medical benefit of these 2,000 insured persons are paid over direct to the "Institution," amounting to some £900 a year. This is the sum which would be available for providing medical attendance and drugs for the 2,000 insured, with extra moneys available for anæsthetics, fractures, operations, and so forth. Be it remarked that part of this sum comes from the State (*i.e.*, from taxation), and the whole of it is from the revenues of a public authority, State created and controlled.

How does the "Institution" spend this £900? It employs two whole-time doctors at salaries of £350 and £250 respectively, who have to attend not only the 2,000 workmen, but also their 4,000 wives and children. In other words, these two unfortunate practitioners get less pay between them than the panel allowance for 2,000 patients would amount to, and have three times that number to look after! Moreover, since the majority of these are women and children, they must have far more than three times the amount of work to do; and, in addition, are completely under the thumbs of taskmasters as hard and grinding as trade unionists alone know how to be. In other words, they are being abominably sweated.

The thoughts that naturally suggest themselves are, What manner of men can these be who accept such servitude? Why have they sold themselves for such a miserable price? What kind of professional reputations and ability do they possess? These questions the *Lancet* answers only inferentially by "giving up" the riddle altogether. It is also stated that many of the workmen themselves are finding out that membership of this "institution" (and of many similar organisations in other parts of Wales) is not all that they expected. In other words, that sweated labour is seldom the best value for money. It is also asserted that the large membership which these institutions have achieved is partly due to fraud, and partly to intimidation. Protests to the local Insurance Committees and to the Welsh Insurance Commission are useless; there is hardly a pretence of investigation, and no prospect of redress.

This super-panel system fairly outdoes the worst that can be said against the ordinary panels themselves. The profession of medicine has indeed sunk low when even two or three can be found willing to work on terms like these. But, apparently, the supply is by no means negligible, for we hear of one town (Blaenavon) where five doctors are thus employed by one "institution." Panellers will please note what things are coming to in Wales, whence England seems to be so largely governed nowadays.

